# Assessment of Viticultural Biodiversity: Recovery of Indigenous Grapevine Genotypes from Ancient Vineyards of El-Kantara (*Calceus Herculis*) in Algeria

**DOI:** 10.3390/plants15091381

**Published:** 2026-04-30

**Authors:** Hanane Achour, Ziane Laiadi, Wahiba Yahiaoui, Valentina Fantin, Irene Olivotto, Daniele Migliaro

**Affiliations:** 1 Laboratory of Genetic, Biotechnology and Valorization of Bioresources (LGBVB), University of Biskra, BP 145 RP, Biskra 07000, Algeria; hanane.achour@univ-biskra.dz (H.A.); ziane.laiadi@univ-biskra.dz (Z.L.); wahiba.yahiaoui@univ-biskra.dz (W.Y.); 2 Council for Agricultural Research and Economics, Research Centre for Viticulture and Enology, Viale XXVIII Aprile 26, 31015 Conegliano, Italy; valentinafantin204@gmail.com (V.F.); daniele.migliaro@crea.gov.it (D.M.)

**Keywords:** *Vitis vinifera* L., El-Kantara (*Calceus Herculis*) vineyards, ampelographic descriptions, genetic diversity, SSR markers, conservation

## Abstract

The vineyards of El-Kantara (*Calceus Herculis*, Algeria) have an ancient viticultural tradition that has never been scientifically documented. This study aims to evaluate the genetic and phenotypic diversity of this region to preserve native grapevine genetic resources. A combined approach of simple sequence repeat (SSR) markers and ampelographic characterization based on 35 Organisation Internationale de la Vigne et du Vin (OIV) descriptors was applied to 51 grapevine cultivars. Genetic analysis revealed moderate diversity, identifying eight international Mediterranean varieties, four known Algerian cultivars, and six novel genotypes with proposed names ‘Aïn Taher’, ‘Ineb Ganteri’, ‘Datté Ganteri’, ‘Seouikiya’, ‘Bayedha d’El-Kantara’, and ‘Ineb ElDjebel’. Ampelographic analyses revealed significant phenotypic variation, with principal component analysis (PCA) explaining 77% of the total variance, primarily driven by vein length and sinus shape. Cluster analysis demonstrated strong alignment between molecular and morphological data, grouping the novel genotypes into distinct morphological categories. These findings highlight a unique and previously undocumented genetic heritage in El-Kantara’s vineyards and underscore the need for a national strategy to conserve and promote Algeria’s native grapevine resources, ensuring their preservation for future viticultural and breeding programmes.

## 1. Introduction

Grapevine (*Vitis vinifera* L.) is a species of great economic and cultural importance, especially in Mediterranean regions where it has been cultivated for thousands of years [1]. The Maghreb region was particularly suited to this type of agriculture due to its favourable conditions. The Phoenicians played a key role in the development of arboriculture in the region, aware of this potential: even though grapevines, olives, and figs are native varieties, they probably began cultivating them in an organized way [2,3]. They also introduced oriental varieties, grafted wild trees, practiced caprification on fig trees, and applied the sophisticated techniques of arboriculture that they had mastered for centuries [4]. In Africa, as in their homeland, they began to produce wine, which testifies to their agricultural expertise and to the influence they exerted in the region [5]. In the Maghreb countries, particularly Algeria, viticulture has been characterized historically by a rich diversity of local grape varieties [6]. Since the expansion of this crop during the Phoenician, Carthaginian and Roman periods, this practice has become a source of income, like the olive tree and the fig tree [7]. Later, the introduction of table grapes from the Middle East to North Africa was a Muslim act [8]. Further, during the period of French colonization, Algerian viticulture experienced a very strong development of vines for obvious agro-economic reasons, but on the other hand, table viticulture did not receive any particular attention. At the time, the predominant vine was the ‘Chasselas’, whose production was mainly intended for export [9].

However, Algeria’s viticultural potential is considerable, although still poorly documented, particularly regarding local varieties and their relationship with Mediterranean and international grape varieties [10,11]. According to the 2019 report ‘The State of Biodiversity for Food and Agriculture in Algeria’ published by the Ministry of Agriculture, Rural Development and Fisheries, in collaboration with the Food and Agriculture Organization of the United Nations (FAO), the persisting degradation of Algeria’s plant heritage, particularly the loss of biodiversity, is a cause for concern. The report indicates that more than 95% of the varieties recorded in Algeria have disappeared [12]. However, the risk of genetic erosion for this species is high due to the lack of systematic protection and conservation measures at the national level, which has led to the rapid decline of local varieties. In addition, the national grape landscape is currently dominated by non-native varieties. This situation is the result of the introduction of exotic varieties, mainly from Europe, which have replaced local varieties [13]. Therefore, to understand the origin and diversity of grape varieties and to preserve this rich heritage, the genetic characterization of grape varieties is essential.

This study will contribute to the discovery and conservation of local grape varieties grown in unmanaged sites such as El-Kantara. The oasis of El-Kantara takes its name from a bridge built by the Romans (*Calceus Herculis*) [14]. Roman ruins are mainly found upstream from the oasis and in Dachera, which was built on a conglomerate sandstone bank overlooking the river (Oued Elhay) and a palm grove, representing the first settlement in this area. Another village, named ‘le Grégeur’, was built beyond the palm grove on the opposite side of the Oued Elhay [15,16]. According to historical studies of the region, *Calceus Herculis* was a small town with a diverse population, including Semites, Punics, Syrians, and notably Palmyrenes, who settled there in the latter half of the 2nd century AD, alongside Berbers [17,18]. El-Kantara was thus presented as a society of Romanized Berber villagers and Semitic communities. It was also a centre of colonization and agricultural development, particularly for crops such as wheat and grapevines [17,18]. In 682 AD, Okba Ibn Nafaa and his army settled in southern Algeria along the riverbanks, founding the first settlements thanks to the topography of the area and the natural fault, which offered security, a permanent water source, and access to oases. One of these settlements was later named ‘Dachra Dahraouia’. During the second invasion, in 1048 AD, Berber and Arab tribes settled in the south-east of Dachra, in ‘le Grégeur’, and gave rise to another village called ‘BorAbbas’ [15].

Arab (Muslim) influence during this period cannot be ruled out, either through the introduction of Asian varieties from their sphere of influence and, later, following the withdrawal from the Iberian Peninsula, through the use of Spanish varieties [19]. This influence was associated with a focus on the production of fresh grapes and raisins [10,11,12,13,14,15,16,17,18,19,20]. The Muslim invasion thus marked the end of wine production, but not to the cultivation of vines to produce table grapes and raisins [10,11,12,13,14,15,16,17,18,19,20,21].

This study aims to characterize the diversity of local grapevine varieties by combining detailed ampelographic descriptors and molecular markers (SSR). This integrated approach will provide a better understanding of the diversity and genetic relationships among Algerian grapevine cultivars, while laying the foundations for a national strategy to conserve and promote local cultivars.

## 2. Materials and Methods

### 2.1. Plant Materials

Fifty-one local cultivars were analyzed in this study (Table 1).

**Figure 1:**
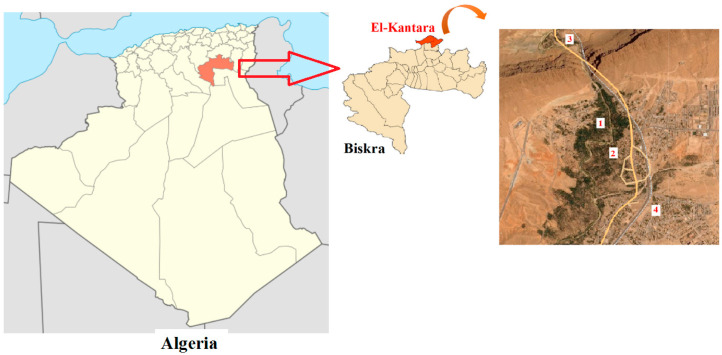
Geographical locations in the El-Kantara commune (Algeria) where genotypes were collected. 1: Dachera; 2: Bourj El-Ghoula (Greuger); 3: El-Ghousse; and 4: El-Faidhe. The list of the geographic coordinates of the cultivar is provided in Supplementary Table S1.

These cultivars were collected directly from different areas of the El-Kantara commune (Figure 1), in particular: El-Faidhe, El-Ghousse, Bourj El-Ghoula (Greuger) and Dachera, the last two being the oldest sites in this commune, where it is easy to find elderly vines (over 90 years old).

**Figure 2:**
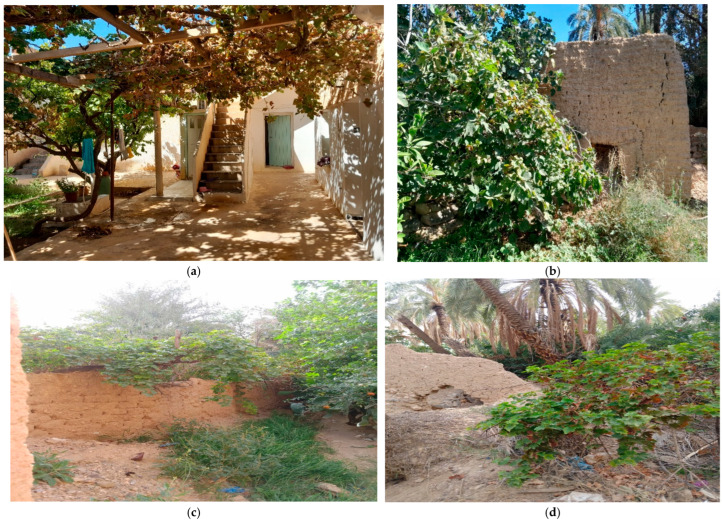

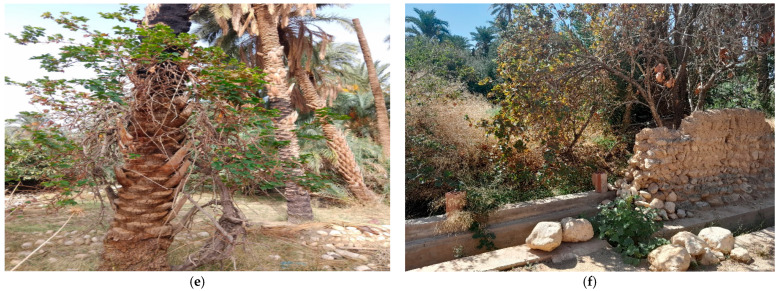
Vines growing in different parts of the oasis: (**a**,**b**) vines growing inside an ancient traditional house in El-Kantara; (**c**,**d**) vines climbing the wall of a forest; and (**e**,**f**) vines climbing palm and pomegranate trees.

Most of these vines grow in natural areas on the riverbanks or in palm groves, while others have been planted close to an ancient abode (Figure 2).

However, it is interesting to note that the names of grape varieties have been derived from distinctive morphological features linked to the shape and colour of the berries or distinctive characteristics such as the musky aroma. While some varieties were named according to their geographical origin, many accessions (especially those collected from abandoned vineyards) had no name and were therefore labelled as ‘Anonymous’.

In addition, a large number of varieties sharing the same name exhibited different genotypes. This discrepancy is attributable to the lack of formal ampelographic documentation for these varieties, which are specific to the Aures region and the northern Sahara oases. This provides clear evidence of the need for genetic characterization in order to fully understand and effectively conserve the diversity of the El-Kantara vineyards.

### 2.2. DNA Extraction and SSR Analysis

For each sample, wood cambium tissues were lyophilized and then ground to a fine powder using a Tissue-Lyser II instrument (Qiagen, Hilden, Germany). DNA was extracted from 20 mg of powder using a Qiagen DNeasy Plant mini kit (Qiagen, Hilden, Germany), according to the manufacturer’s protocol. The quality of the extracted DNA was verified by 1% agarose gel electrophoresis. A NanoDrop 1000 spectrophotometer (Thermo Fisher Scientific, Waltham, MA, USA) was used to determine the concentration and purity of the DNA.

Twelve SSR loci were used to characterize the cultivars studied: the nine proposed as common grapevine markers for international use within the framework of the Grapegene06 European project (VVS2, VVMD5, VVMD7, VVMD25, VVMD27, VVMD28, VVMD32, VrZAG62, VrZAG79), plus VMC6E1, VMC6G1 and VMCNG4b9 (Vitis Microsatellite Consortium). The SSR markers, each with a high polymorphism, have been used by the European consortium GrapeGen06 [22] and have often been used to identify the cultivars studied by comparing genotyping data obtained from published genotypes or public databases [23,24,25,26]. PCR reactions were performed using forward primers labelled with fluorescent dyes (6-FAM, PET, VIC, or NED); two multiplex panels of fluorescent-labelled microsatellite loci were used. Simultaneous PCR amplifications were conducted in a final volume of 20 μL containing 1 × PCR reaction buffer, 10 ng of genomic DNA, 0.2 mM of each dNTP, 2 mM MgCl2, and 1.5 U Taq DNA polymerase (Thermo Fischer Scientific, Waltham, MA, USA). Depending on the locus, primer concentrations ranged from 0.11 to 0.48 μM. The following profile was used: a hot start of 95 °C for 5 min, 30 amplification cycles of 45 s at 95 °C, 1 min at 55 °C, 30 s at 72 °C and a final extension step of 30 min at 72 °C. PCR products (0.5 μL) were mixed with 9.35 μL formamide and 0.15 μL GeneScan™ 500 LIZ Size Standard (Thermo Fisher Scientific, Waltham, MA, USA). Capillary electrophoresis was conducted in an ABI 3130xl Genetic Analyzer (Thermo Fisher Scientific, Waltham, MA, USA). Allele calling was performed using GeneMapper 4.0 software (Thermo Fisher Scientific, Waltham, MA, USA). Allele sizes were recorded in bp, and genotypes with a single peak at a given locus were considered homozygous.

Identification was performed by comparing the obtained SSR profiles with the CREA Viticulture and Enology molecular database, which currently contains about 8000 unique profiles and is constantly updated (partly published in the Italian Grapevine Catalogue [27]), literature information and the *Vitis* International Variety Catalogue [28].

### 2.3. Statistical Analysis on Genotyping Data

Statistical analyses were performed using several specialized software tools. Cervus 3.0 software [29] was used for parentage analysis when progenitors were unknown [30]. Genetic diversity parameters, including the number of different alleles (No alleles), the effective number of alleles (Ne alleles), observed (Ho), expected heterozygosity (He), Hardy–Weinberg equilibrium (HW), and probability of null alleles (F null), were estimated using the GenAlEx 6.5 software [31].

### 2.4. Ampelographic Characterization

The study focused on the ampelographic characterization of mature leaves from the newly identified grapevine varieties recovered from the El-Kantara region, Algeria. A total of 11 leaf samples were collected from each variety during the 2021–2022 growing season, following the descriptor methodology of the International Organisation of Vine and Wine [32]. For each genotype, 11 mature leaves were collected from a single vine to represent biological replicates. Measurements obtained from these leaves were averaged and used for statistical analyses. Leaves were sampled from healthy and mature leaves located on the middle portion of the shoots (4th to 6th node from the base), ensuring that leaf morphology was fully developed. The vines were located in traditional vineyards and exposed to the typical climatic conditions of the El-Kantara region.

For each leaf, the following traits were measured and recorded: 17 quantitative traits (OIV 601-OIV 617), and 18 qualitative traits (OIV 067, OIV 068, OIV 070, OIV 071, OIV 076, OIV 079, OIV 080, OIV 081-1, OIV 082, OIV 083-2, OIV 084, OIV 085, OIV 088, OIV 089, OIV 090, OIV 091, OIV 093, and OIV 094).

All measurements were performed using ImageJ software 1.54r [33]. Qualitative characteristics were recorded by visual inspection and classified according to the standard OIV code system.

### 2.5. Statistical Evaluation of Ampelographic Data

Principal component analysis (PCA) of the quantitative traits recorded in the study was performed using PAST software 5.3 [34]. Correlation analysis, based on the Pearson correlation coefficient, was applied to determine the relationships between the observed traits. Cluster analysis was conducted using Ward’s method on both quantitative and qualitative traits, applying the coding method based on the grapevine descriptor guidelines from OIV, in order to assess the phenotypic relationships among the studied cultivars.

## 3. Results and Discussion

### 3.1. Genotyping of El-Kantara Varieties

The twelve SSR markers identified 18 different molecular profiles among the 51 analyzed samples (Supplementary Table S2). The genetic profiles obtained were then compared with the 6354 references available in the *Vitis* International Variety Catalogue *V*IVC [28], as well as with the 8000 genetic profiles stored in the database of the Council for Agricultural Research and Economics (CREA Viticulture and Enology), and with SSR genotypes reported in the literature. Based on these comparisons, SSR data have been defined for specific genotypes (Table 1).

Analysis of microsatellite profiles revealed a significant presence of non-native varieties originating from the Eastern Mediterranean, including ‘Dabouki’ and ‘Afus Ali’, from North African countries such as Tunisian ‘Rassegui’ and Moroccan ‘Taferielt’, and from Eastern Europe, in particular the Greek ‘Muscat of Alexandria’. In addition, varieties of Western origin were detected, such as the French cultivars ‘Danugue’ and ‘Dattier de St. Vallier’ and the American cultivar ‘Cardinal’.

This diversity confirms the historical data that document the introduction of several grapevine varieties from different regions into El-Kantara over time. However, several international varieties are locally known under dialectal names. These local denominations are probably influenced by nurseries that supply these varieties or by farmers who adopt familiar or descriptive names.

In the present study, the genotype ‘Dabouki’ was the most abundant in the community of El-Kantara, represented by eleven accessions. These findings are similar to those of Rahali et al. [8], who reported that the Babar region (province of Khenchela, Algeria) is mainly cultivated with ‘Dabouki’. As this region is geographically not far from the study area, which is part of the Aures zone, it can be inferred that ‘Dabouki’ is the most cultivated variety in both the Aures region and the northern Sahara. Its high frequency indicates its importance as an emblematic cultivar for the whole region, suggesting a shared cultural and historical heritage across borders.

The second most represented variety, ‘Rassegui’, was identified in four samples. This cultivar is reported to be the widely cultivated variety in all Tunisian grape-growing regions.

Moreover, two samples were identified as belonging to the Lebanese variety ‘Afus Ali’, one of the most widespread table grape cultivars in Mediterranean countries, with 67 registered accessions and 253 reported synonyms [28], among which ‘Dattier de Beyrouth’ is the most frequently cited [35]. In the studied area, ‘Afus Ali’ is also locally known as ‘Mestateouile’, because of its large, elongated berries resembling dates.

However, varieties of putative French origin were also detected, including ‘Danugue’ [36] represented by three samples, and a single accession of ‘Dattier de St. Vallier’. In the studied area, the latter variety is locally known by the synonym ‘Kahela’, which means ‘black’. Despite sharing the same genotype, these two accessions exhibited different berry colours. This colour variation can be caused by a somatic mutation, and this type of event is common in table grapes [37].

According to our analysis, two samples were identified as ‘Muscat of Alexandria’, a hardy variety tolerant to drought and hot climates but sensitive to high humidity [38]. This white variety is widely cultivated throughout the Mediterranean basin [39], as well as in South Africa, California, Australia and South America, with more than 200 registered synonyms [38].

Among the identified varieties, one accession corresponded to the ‘Cardinal’ genotype. In the studied area, this variety is locally known as ‘Datté’ because of the shape and colour of its berries, which are long and dark and resemble dates.

Two samples were identified as ‘Taferielt’, a Moroccan variety characterized by chlorotype D, also known as ‘Farana Noir’ [39]. The first ampelographic characterization of ‘Farana’ from the Blida region (Algeria) was reported by Pulliat [40]. While Laiadi et al. [41] previously applied the name ‘Farana Noir’ to a sample that was genetically identified as ‘Oul b’ozeghur’ (VIVC 8844), our data confirm that the true ‘Farana Noir’ is genetically identical to ‘Taferielt’. This correspondence also extends to the variety locally known as ‘Azbib’ in the Aures region [8], establishing that ‘Farana Noir’, ‘Azbib’, and ‘Taferielt’ all share the same genotype. This case highlights the fundamental importance of precise genetic investigations in resolving the nomenclatural confusion and historical misidentifications caused by local traditions and previous analytical errors (Table 2).

**Table 2:** SSR correspondences for the varieties ‘Farana Noir’, ‘Azbib’ and ‘Taferielt’ in the literature.

Sample	VVS2	VVMD5	VVMD7	VVMD25	VVMD27	VVMD28	VVMD32	VrZAG62	VrZAG79	VMC6 E1	VMC6G1	VMCNG4b9
‘Farana Noir’ (‘Oul b’Ouzguer’) in [42]	137	242	249	241	184	258	252	200	259	**/**	**/**	**/**
	143	242	253	255	195	260	262	204	259	/	/	/
‘Azbib’ (‘Taferielt’) in [8]	133	240	249	239	195	243	252	194	257	141	191	176
	135	242	249	241	195	260	272	204	257	165	191	176
‘Taferielt’ in [43]	133	240	249	239	195	243	252	/	/	/	/	/
	135	242	249	241	195	260	272	/	/	/	/	/
‘Farana Noir’ (‘Taferielt’) in our study	133	240	249	239	195	243	252	194	257	141	191	176
	135	242	249	241	195	260	272	204	257	165	191	176

In the studied area, autochthonous Algerian grapevine varieties such as ‘Amokrane’, ‘Ahmeur Bou Ahmeur’, ‘Louali’ and ‘Amer Bouamar’ were identified. According to Laiadi et al. [42], ‘Amokrane’ is one of the most popular varieties in Algeria. In the region of interest, it is called ‘Baydha’, which means ‘white’ in the local dialect. ‘Ahmeur Bou Ahmeur’ is also a traditional Algerian variety that has spread all over the world [44]. According to Laiadi et al. [42], Borrego et al. [45], Ibáñez et al. [46], and Zinelabidine et al. [47], the same genotype is cultivated in Spain under different names like ‘Royal Gordo’ and ‘Teta de Vaca’, and is also known as ‘Flame Tokay’ [48]. This diffusion is probably the result of cultural and material exchanges between North Africa and the Iberian Peninsula over many centuries.

Moreover, the ‘Louali’ genotype was also identified in the studied area. A clear genetic difference between ‘Louali’ and ‘Amokrane’ was observed, meaning that the two varieties are not synonymous. This result is similar to that obtained in Khouni et al. [49]. However, in the study by Laiadi et al. [42], the accessions named ‘Amokrane’ and ‘Louali’ showed identical SSR profiles. In fact, the accession named ‘Amokrane’ in that study corresponded genetically to ‘Louali’, while accessions named ‘Amokrane’ were erroneously assigned to other cultivars (‘Ahmed draa el Mizen’, ‘Amellal’, ‘Aneb Kabyle’ and ‘Tinesrine’), highlighting the complexity of local nomenclature and frequent misidentifications.

In addition, a local variety named ‘Amer Bouamar’ was identified: this variety is known in the studied area under the common names ‘Zitouna’ and ‘Kahela’, and was first documented in Bouzina (Algeria) by Yahiaoui et al. [21].

In the present study, new grapevine genotypes were discovered and documented. These varieties, indicated with popular names (six different genetic profiles corresponding to sixteen samples), had not been previously reported in the scientific literature and did not correspond to any profile recorded in the CREA-VE database or in the *Vitis* International Variety Catalogue [28]. These results demonstrate the presence of a unique and undocumented genetic diversity within the grapevine gene pool in Algeria, more specifically in the El-Kantara region. The accessions were mainly named according to their geographical origin (e.g., ‘ElDjebel’, referring to the Metelili mountains), morphological characteristics such as berry shape or colour (e.g., ‘Khadhera’, which means ‘green colour’), or specific characteristics, such as ‘Seouikiya’, which refers to its distinctive musky aroma and sweet taste.

Among these newly identified varieties, ‘Genotype 1’, represented by six grapes, was proposed to be named ‘Aïn Taher’, and ‘Genotype 6’ was proposed as ‘Ineb ElDjebel’. According to the SSR profile data comparisons (Supplementary Table S2), it could be noted that both genotypes are related to the variety ‘Danugue’, as they share most of the alleles with this variety across the twelve SSR loci. The two genotypes also shared six identical loci and exhibited a high level of allelic similarity.

Furthermore, ‘Genotype 2’, represented by five accessions, was proposed to be named ‘Ineb Ganteri’. ’Genotype 4’, proposed as ‘Seouikiya’, showed a potential parent–offspring relationship with the variety ‘Taferielt’, since they share eleven to sixteen alleles per locus across the twelve SSR markers. For ‘Genotypes 3’ and ‘Genotype 5’, the names ‘Datté Ganteri’ and ‘Bayedha d’El-Kantara’ were respectively proposed. These two varieties exhibited a strong parent–offspring relationship with ‘Louali’ and shared almost all alleles across the twelve SSR loci analyzed.

These newly identified genotypes are likely to represent minor native cultivars of local importance, characterized by valuable traits such as fruit quality and adaptation to local environments. According to the SSR profiles obtained in this study, ‘Louali’ and ‘Taferielt’, which are involved in many parent–offspring relationships, could have played a role in the diversification of local grapevine germplasm.

### 3.2. Genetic Diversity

A total of 87 different alleles were detected across the 12 SSR loci analyzed, confirming an overall acceptable level of genetic diversity between the 51 accessions studied (Table 3), with an average of 7.250 alleles per locus.

The number of different alleles per locus (No) ranged from five (VVMD27) to nine (VVS2 and VrZAG79), while the effective number of alleles (Ne) varied from 3.340 (VVMD25) to 6.612 (VVS2), with a mean Ne of 4.578. These values are comparable to those reported by Rahali et al. [8] for grapevine accessions from the Babar region (Ne = 4.727 and No = 7.000), but lower than those observed by Khouni et al. [49], who reported higher allelic richness (149 alleles across 12 loci; mean No = 12.42 and mean Ne = 7.00) in an extended taxonomic set (M’zej Edchiche de Sekikda collection) including multiple species of Vitis. This confirms that taxonomically broader sampling contributes to a higher number of alleles.

The mean observed heterozygosity (Ho) and expected heterozygosity (He) were 0.806 and 0.771, respectively, indicating a high level of polymorphism and substantial genetic diversity. Expected heterozygosity ranged from 0.701 (MD25) to 0.849 (VVS2). The average expected heterozygosity (77%) was lower than that reported for other Algerian collections with 85% [49] and 86% [50], as well as for some international collections, such as the Brazilian with 85% [51] and the Georgian with 80.7% [52]. However, it was comparable to values reported for Turkish (75%) [53], Armenian (78.9%) [54], Moroccan (76%) [43], and Tunisian (75.0%) [55] collections.

The lowest observed heterozygosity (Ho = 0.667) was found at locus VVMD25, and the highest value (Ho = 1000) was observed at locus VVMD5. Overall, the mean Ho was 0.806. These values are higher than those reported in some previous grapevine studies, such as 69.3% in the central Mediterranean for 295 genotypes [56], 71.96% in North Africa (Maghreb region) for 181 genotypes [57], 74.2% in Central Asia for 1378 genotypes [58] and 75% in Brazil for 410 genotypes [59].

The inbreeding coefficient F(Null) was negative for 9 of the 12 SSR loci, while weak positive values were detected for VVMD25, ZAG79 and VMC6E1, where He exceeded Ho. This pattern suggests the possible presence of null alleles at these loci, potentially due to mutations affecting primer binding sites [60]. Most loci did not deviate significantly from the Hardy–Weinberg equilibrium, indicating a well-mixed population, with the exception of ZAG79 and VMC6G1.

The highest Shannon index was observed at locus VVS2 (2.006) and the lowest at locus VVMD27 (1.367), with an average of 1.673. The Shannon index reflects the level of polymorphism and indicates moderate to high allelic diversity across loci.

The probability of identity (PI) varied among loci, with the highest value for VVMD25 (0.140) and the lowest for VVS2 (0.041), indicating a good discriminatory power for most markers. The cumulative PI across the 12 loci was estimated to be 8.04 × 10^−14^, suggesting that the chance of two different varieties having identical genetic profiles is extremely low. This PI value is higher than those reported for Algeria (7.35 × 10^−18^ in Khouni et al. [49]; 3.4 × 10^−15^ in Laiadi et al. [42]), and southern Umbria, central Italy (1.6 × 10^−15^ in Zombardo et al. [61]), but lower compared to the PI of Bulgaria (1.2 × 10^−8^ in Hvarleva et al. [62]) and Spain (9.9 × 10^−12^ in Ibáñez et al. [46]).

The reliability of microsatellites for varietal identification depends on the probability of identity (PI) [63]. SSRs that are effective in genotypic discrimination have high levels of Ho and low values of PI [64,65]. In this study, VVS2 showed high levels of diversity in terms of expected heterozygosity (Ho = 0.889; higher than the mean Ho of 0.806) and the lowest values of PI (0.041). However, observed heterozygosity for VVMD5 and VVMD28 was higher than expected (Ho > He), which could be a sign of selection. In contrast, VVMD25, ZAG79 and VMC6E1 showed a slight positive deviation from F null values, suggesting low but detectable inbreeding. These loci need further investigation to clarify the underlying causes of these deviations.

Overall, the diversity data show an acceptable genetic diversity among the 51 genotypes studied, with a balanced genetic structure and low levels of inbreeding.

### 3.3. Phylogenetic Dendrogram

**Figure 3:**
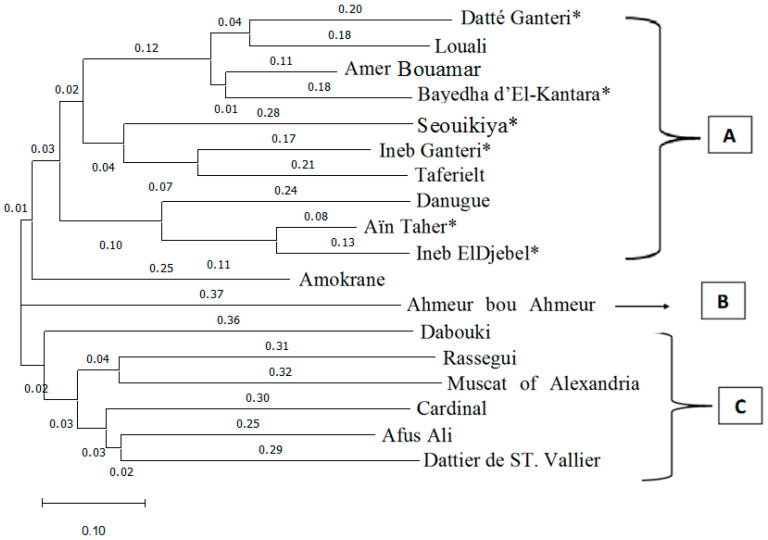
Dendrogram illustrating the genetic relationships between 18 genotypes for the 12 microsatellite loci. The tree was obtained using the NJ algorithm. Three main groups were identified: group A includes all autochthonous varieties and the newly identified genotypes from the El-Kantara region; group B comprises a single Algerian autochthonous variety, ‘Ahmer Bou Ahmeur’; and group C comprises a heterogeneous set of genotypes corresponding mainly to internationally known cultivars. The scale bar represents the genetic distance. *: newly identified varieties.

The phylogenetic dendrogram represents the genetic relationships between 18 genotypes based on SSR analysis and clearly shows a significant level of genetic polymorphism, allowing the identification of three different groups of varieties (A, B, C), respectively containing eleven, one and six varieties. Each group was further divided into several subgroups (Figure 3).

Group A includes all native varieties and newly identified genotypes in the El-Kantara region, whose NJ similarity indices range from 0.03 to 0.25. This group is structured in four clearly distinct clades.

The first subgroup comprises four varieties that show very close and significant phylogenetic relationships. Notably, the two newly identified genotypes, ‘Datté Ganteri’ and ‘Bayedha d’El-Kantara’, display a strong genetic relationship with ‘Louali’ and ‘Amer Bouamar’, sharing almost all the alleles of the 12 SSR loci analyzed. These relationships are confirmed by the phylogenetic tree, in which the NJ similarity indices range from 0.18 to 0.20 between the two varieties ‘Datté Ganteri’ and ‘Louali’, and from 0.11 to 0.18 between ‘Amer Bouamar’ and ‘Bayedha d’El-Kantara’.

The three varieties ‘Ineb Ganteri’, ‘Seouikiya’ and ‘Taferielt’ form a single clade expressing their strong genetic link, confirming the parental relationship previously mentioned.

The third subgroup is represented by three closely related varieties, ‘Aïn Taher’ and ‘Ineb ElDjebel’ together with the French variety ‘Danugue’ (VIVC variety number 3425). However, other studies [66] have shown that ‘Danugue’ is related to a Spanish variety ‘Breval negro’. Despite its distribution in Algeria is notably localized, recent studies failed to identified ‘Danugue’ in the north-east (in the M’zej Edchiche vine germplasm collection in Skikda), and in the north-west (in the Teghennif experimental station in Mascara) [41,42,49,50]. By contrast, Rahali et al. [8] and Yahiaoui et al. [21] reported that the local variety ‘Babari’ also has a strong genetic link with ‘Danugue’, which they identified only in the Aures region (Babar commune in Khanchela and Batna region). In addition, according to this study, the analysis of the SSRs places the ‘Danugue’ at the centre of the cluster of newly identified local varieties, raising questions about its true geographical origin. Although traditionally considered a French cultivar, its strong genetic relationship with several local Algerian varieties and its restricted occurrence in the Aures region suggest that this area may represent its centre of origin. The subsequent diffusion of this cultivar from North Africa to the Iberian Peninsula and then to Central Europe (such as France) may have occurred during historical periods of Muslim presence in southern Europe. This hypothesis agrees with the findings of Laiadi et al. [42], who reported identical genotypes between certain Algerian and Iberian cultivars, reflecting the long history of interaction and exchange between the two regions.

The fourth subgroup is represented by the variety ‘Amokrane’, which is described in the ampelographic literature as an Algerian cultivar [42]. Its clustering with newly identified cultivars suggests a close genetic relationship.

Group B is represented by the only autochthonous Algerian variety ‘Ahmeur Bou Ahmeur’.

Finally, Group C includes a heterogeneous set of genotypes of supposedly different origins. These include ‘Dabouki’, ‘Rassegui’, ‘Muscat of Alexandria’, ‘Cardinal’, ‘Afus Ali’ and ‘Dattier de St. Vallier’, with NJ similarity indices ranging from 0.02 to 0.39.

### 3.4. Phenotypic Diversity Among Studied Genotypes

#### 3.4.1. Correlations Among Variables

**Figure 4:**
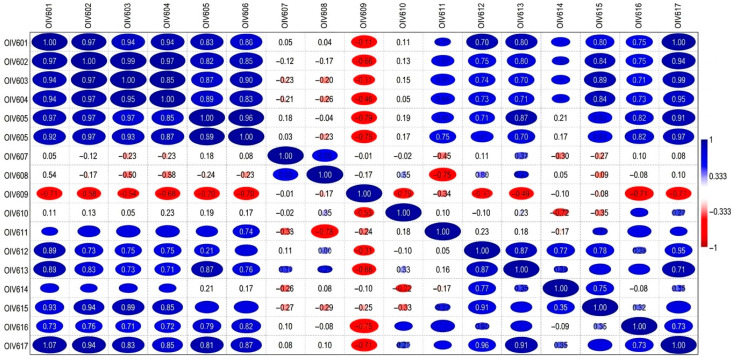
Correlation matrix of 17 ampelometric traits among novel grapevine varieties from El Kantara (Biskra, Algeria).

The ampelometric dataset revealed significant correlations among the studied variables (Figure 4).

This simple correlation analysis facilitated the identification of relationships between morphological traits, as also reported by Bodor-Pesti et al. [67]. However, only a limited number of studies in the literature emphasize the correlation between key mature leaf characters based on OIV descriptors. In line with this, Susaj et al. [68] identified strong correlation relationships among main mature leaf traits, such as length of main veins (OIV 601, OIV 602, OIV 603, OIV 604, OIV 611), length of petiole (LP), length of petiole sinus to upper and lower lateral leaf sinuses (OIV 605 and OIV 606), and angle size (OIV 607 and OIV 608). On the other hand, Bodor et al. [69] found a significant correlation between the two halves of the leaves (except for OIV 608). According to our results (Figure 4), very strong positive correlations were observed among OIV 601 to OIV 606 (r = 0.88 to 0.98), suggesting that these traits are genetically related and may be influenced by similar environmental factors or growth conditions, as partially reported by Bodor et al. [70] and Chitwood et al. [71]. Notably, OIV 617 also showed strong positive correlations with the early numbered descriptors (r = 0.82 to 0.94). On the other hand, moderate to strong positive correlations (r = 0.62 to 0.89) appeared among OIV 612 and OIV 613, as well as for OIV 615 and OIV 616, with the aforementioned features (r = 0.54 to 0.94), whereas OIV 611 showed moderate positive correlations (r = 0.46 to 0.75). In contrast, several variables exhibited negative correlations, indicating inverse relationships among morphological features. For instance, negative correlations were recorded among OIV 609 and the vein lengths descriptors (OIV 601 to OIV 604; r = −0.32 to −0.52) as well as with leaf sinuses (OIV 605 and OIV 606; r = −0.67 and −0.66, respectively). Furthermore, OIV 609 was negatively correlated with OIV 616 and OIV 617 (r = −0.73 and −0.50, respectively). This pattern suggests that as the angle between veins N3 and N4 (represented by OIV 609) increases, the lengths of veins N1, N2, N3, and N4, as well as the number of teeth and the distance from the petiole sinus to the upper and lower lateral leaf sinuses, tend to decrease. Correspondingly, Welter et al. [72] highlighted that leaf angles are strongly associated with the degree of opening or overlapping of the leaf sinus, suggesting an accumulation of morphogenetic factors, particularly those influencing the depth of the leaf sinus. Moreover, strong negative correlations were observed between OIV 610 and OIV 614 (r = −0.91), and between OIV 610 and OIV 615 (r = −0.68). Other examples of negative correlations include those between OIV 608 and OIV 611 (r = −0.76). Lastly, OIV 607, OIV 610 and OIV 614 showed predominantly weak or negligible correlations with most other variables (correlation values close to 0, shown in white or pale colours in Figure 4), suggesting limited relationships with the remaining traits.

#### 3.4.2. PCA of Grape Genotypes Based on Quantitative Traits

**Figure 5:**
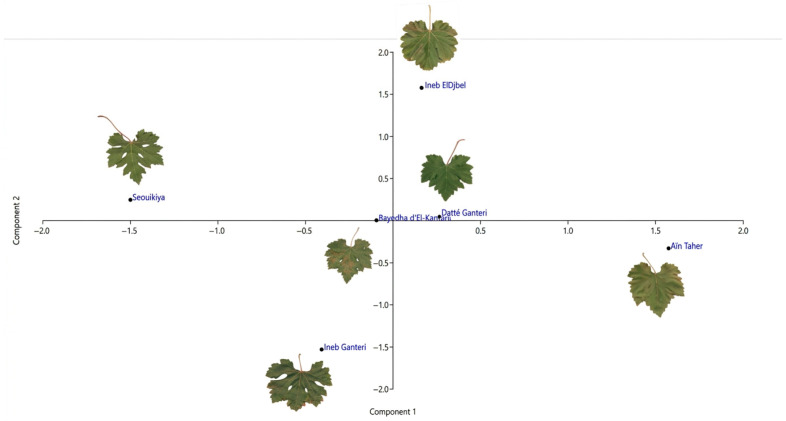
Two-dimensional scatter plot of the first two principal components (PC1 and PC2) showing the distribution of novel grapevine genotypes based on 17 ampelometric leaf traits.

The results of the principal component analysis (PCA) performed for the ampelographic characterization of six novel grapevine genotypes recovered from El-Kantara (Biskra), based on 17 OIV characters (Supplementary Table S3.a), are shown in Figure 5.

PCA is widely applied in phenotypic description and morphological characterization of many fruit species and cultivars, noting olive (*Olea europea* L.) [73], fig (*Ficus carica* L.) [74], date palm (*Phoenix dactylifera* L.) [75], as well as grapevine studies [76,77,78,79,80,81].

The first three components explained a significant portion of the variance in the data (95%). However, the first two PCs were considered to be significant for the studied traits, explaining a total of 77%. Specifically, PC1 accounted for 56.52% of the total variance, capturing the main variations in the dataset, while PC2 explained an additional 20.59%. These results suggest that most of the variability in the leaf morphological traits can be summarized using just two dimensions. The distribution of the varieties is shown in the two-dimensional scatter plot (Figure 5).

The first principal component (PC1) predominantly captured variation associated with vein lengths (OIV 601, OIV 602, OIV 603, and OIV 604), as well as with the lengths of the petiole sinus and the upper and lower lateral leaf sinuses (OIV 605 and OIV 606). Notably, these findings align partially with those reported by Bounab and Laiadi [78] for Algerian grapevines preserved in the germplasm collection of Skikda. More significantly, they closely resemble the results of Yahiaoui et al. [21], which characterized four novel varieties from the Aures region in Algeria. Additional characters contributing to PC1 included length/width of tooth (OIV 612, OIV 613 and OIV 615), number of teeth (OIV 616), and length between the tooth tip of N2 and the tooth tip of the first secondary vein of N2 (OIV 617). As reported by Cunha et al. [82], certain traits, such as the size of the leaf and the length of teeth compared with their width, are discriminant characteristics of the wild grapevine populations located in Portugal. On the other hand, some descriptive criteria, such as OIV 609, OIV 610, and OIV 614, did not show a significant impact in differentiating the varieties along this axis, and as a result, they are more prominent in PC2.

The scatter plot (Figure 5) illustrates the geometric distances between genotypes across all four quadrants, reflecting their dissimilarity based on the measured traits. From the negative to positive values of PC1, the genotypes showed gradual increases in vein length, length of petiole sinus to upper and lower lateral leaf sinuses, length/width of tooth, number of teeth, and length between the tooth tip of N2 and the tooth tip of the first secondary vein of N2. Notably, the genotype ‘Ineb ElDjbel’, located at the top of the plot, displayed high values for vein lengths (OIV 601 to OIV 604), shallower sinuses (OIV 605 and OIV 606), long and wide teeth (OIV 612, OIV 613 and OIV 615), a high number of teeth (OIV 616), and a greater distance between the tooth tip of N2 and the tooth tip of the first secondary vein of N2 (OIV 617). Conversely, the genotype ‘Ineb Ganteri’, located at the bottom of the plot, was characterized by shorter vein lengths, deeper leaf sinuses, shorter and narrower teeth, and a moderate number of teeth.

Along PC2, from negative to positive values, genotypes showed gradually increasing values for angle size (OIV 609 and OIV 610) and length of tooth of N4 (OIV 614). Accordingly, the genotype ‘Seouikiya’, located on the negative side of PC2, exhibits large angles and a shorter tooth length of N4. In contrast, the genotype ‘Aïn Taher’, located on the right part of PC2, showed medium-sized angles and longer N5 vein length.

#### 3.4.3. Cluster Analysis

**Figure 6:**
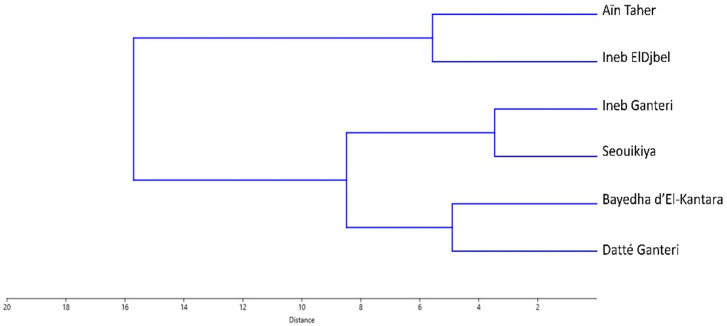
Dendrogram depicting phenotypic relationships among novel grapevine genotypes using Ward’s clustering method.

The resulting dendrogram (Figure 6) clearly discriminated the six novel genotypes into two major clusters, revealing distinct patterns of morphological variation.

Interestingly, the ampelographic classification based on 35 OIV characters (Supplementary Tables S3.b and S3.c), using the Ward’s method, produced a dendrogram that showed strong agreement with the SSR-based clustering and, to some extent, aligned with the PCA results. Moreover, an important observation produced from the SSR-based clustering was that all of the six novel genotypes are grouped along a monophyletic major cluster (Figure 3), in agreement with the findings of Yahiaoui et al. [21] for newly identified varieties specific to the Aures region.

According to the ampelographic dendrogram, the cultivars ‘Ineb Ganteri’ and ‘Seouikiya’ showed the highest degree of similarity (approximately 4 distance units), indicating strong phenotypic resemblance across 12 quantitative characters, including OIV 601-OIV 602, OIV 604-OIV 606, OIV 608-OIV 612, OIV 614 and OIV 617. These two cultivars also shared 17 qualitative OIV descriptors, namely OIV 067, OIV 068, OIV 070, OIV 071, OIV 076, OIV 079, OIV 081-1, OIV 082, OIV 083-2, OIV 084, OIV 085, OIV 088-OIV 091, OIV 093, OIV 094. This pair was subsequently joined by another subcluster formed by the genotypes ‘Bayedha d’El-Kantara’ and ‘Datté Ganteri’, which also displayed high phenotypic similarity, clustering at approximately 5 distance units. Consistently, these varieties are grouped together in the PCA scatter plot, as indicated in Figure 5.

On the other hand, the cultivars ‘Aïn Taher’ and ‘Ineb El Djbel’ formed a cultivar-specific group that merged with the aforementioned cluster at approximately 6 distance units, indicating a higher degree of phenotypic divergence from the other genotypes. This conclusion aligns with the findings of Sabir et al. [83], who observed that in the ampelographic dendrogram, many genotypes tended to fall outside the established groups, likely due to their susceptibility to environmental factors, which can cause variations across different seasons. Upon examination of their ampelographic characteristics, these varieties shared nine quantitative characters (OIV 601, OIV 607-OIV 611, OIV 614, OIV 616 and OIV 617) and 15 qualitative characters (OIV 067, OIV 068, OIV 070, OIV 071, OIV 076, OIV 079, OIV 081-1, OIV 082, OIV 083-2, OIV 084, OIV 088-OIV 090, OIV 093 and OIV 094).

This gradation of similarity within the dendrogram suggests a spectrum of ampelographic characteristics, likely reflecting subtle variations in leaf morphology as described in several studies characterizing grape leaf morphology in Algeria [78], Tunisia [84], Morocco [85], Egypt [86], Lebanon [87], Turkey [88], Greece [89] and Spain [90].

## 4. Conclusions

The recovery and characterization of endangered grapevine cultivars, particularly in historical areas such as El-Kantara, represents an added value that can significantly contribute to the conservation of local biodiversity. The present study sheds light on the remarkable diversity and unique characteristics of the grapevine genotypes cultivated in the El-Kantara region of Biskra (Algeria) by employing a combination of ampelographic and molecular approaches.

The characterization analyses reveal the richness and diversity of the region’s vineyards: a total of 18 genotypes were identified, including international varieties from different countries, most of them Mediterranean (‘Dabouki’, ‘Rassegui’, ‘Farana Noir’, ‘Afus Ali’, ‘Muscat of Alexandria’, ‘Danugue’, ‘Dattier de St. Vallier’ and ‘Cardinal’), four genotypes of Algerian origin (‘Amokrane’, ‘Ahmeur Bou Ahmeur’, ‘Amer Bouamar’ and ‘Louali’) and six genotypes with no correspondence in international databases and never reported, which could represent unique Algerian varieties specific to the El-Kantara region. This work provides the first comprehensive phenotypic and genetic characteristics of these six novel grapevine varieties.

The results revealed significant phenotypic variability among the new genotypes, particularly in traits such as vein length (OIV 601-604), lengths of the petiole sinus and the upper and lower lateral leaf sinuses (OIV 605-606), and tooth dimensions (OIV 612-615), underscoring their potential as markers for varietal discrimination. Moreover, the correlation and PCA analyses provided valuable insights into the relationships and variability among the studied traits, while the cluster analysis highlighted distinct patterns of similarity and divergence among the genotypes.

Importantly, the alignment between molecular (SSR-based) and ampelographic classifications supports the robustness of the combined approach. In particular, the clustering of all the native varieties and the new varieties identified in the El-Kantara region within a monophyletic major group underscores their shared genetic background and local distinctiveness. Overall, this research serves as a pivotal step towards preserving Algeria’s viticultural heritage and underscores the importance of such resources for the advancement of sustainable viticulture.

## Figures and Tables

**Table 1:** List of the 51 vine samples grouped by their genotype. Cultivar code, cultivar name, collection site, meaning of the name in common language, colour of the berries, true-to-type prime name (Correspondence by SSR), and code of the *Vitis* International Variety Catalogue (*V*IVC) are indicated. Berry colour codes: B: blanc (white), N: noir (black), R: rouge (red), RS: rose (pink), and NS: not specified.

Cultivar Code	Cultivar Name	Collection Site	Sample Name Meaning	Berry Colour	Primary Name (Correspondence by SSR)	*V*IVC Code
B4	Anonymous	Bourj El-Ghoula	/	RS	Dabouki B.	3309
B12	Datté	Bourj El-Ghoula	Dattier (in French) or date palm fruit (in English)	B		
D1	Anonymous	Dachera	/	B		
D6	Metateouile	Dachera	Long (in Algerian dialect)	NS		
D15	Muscat	Dachera	Aromatic grape	B		
D18	Chamerakha	Dachera	Arjun (in Algerian dialect), similar to a bunch of date palm fruits	B		
D28	Anonymous	Dachera	/	NS		
F2	Anonymous	El-Faidhe	/	B		
F6	Muscat	El-Faidhe	Aromatic grape	B		
F7	Elhamera	El-Faidhe	Red colour (in Algerian dialect)	RS		
F8	Anonymous	El-Faidhe	/	B		
B3	Anonymous	Bourj El-Ghoula	/	N	Danugue N.	3425
B7	Anonymous	Bourj El-Ghoula	/	R		
D17	Anonymous	Dachera	/	N		
B2	Bayedha	Bourj El-Ghoula	Weight colour (in Algerian dialect)	B	Amokrane B.	431
B8	Anonymous	Bourj El-Ghoula	/	B		
B6	Anonymous	Bourj El-Ghoula	/	R	Rassegui B.	9923
B9	Anonymous	Bourj El-Ghoula	/	NS		
C5	Anonymous	Dachera	/	B		
D20	Anonymous	Dachera	/	B		
D3	Anonymous	Dachera	/	NS	Ahmeur bou Ahmeur B.	140
D10	Anonymous	Dachera	/	B		
D4	Muscat	Dachera	Aromatic grape Sweet taste	B	Muscat of Alexandria B.	8241
D24	Seouikiya	Dachera	(in Algerian dialect)	B		
D11	Anonymous	Dachera	/	NS	Louali B.	24613
D14	Mestateouile	Dachera	Long (in Algerian dialect)	B	Afus Ali B.	122
F4	Mestateouile	El-Faidhe	Long (in Algerian dialect)	B		
D16	Datté	Dachera	Dattier (in French), or date palm fruit (in English)	N	Cardinal N.	2091
F5	Kahela	El-Faidhe	Black colour (in Algerian dialect)	N	Dattier de ST. Vallier B.	3437
F9	Anonymous	El-Faidhe	/	NS	Taferielt N.	12196
F10	Anonymous	El-Faidhe	/	NS		
D12	Zitouna	Dachera	Similar to black olive (in Algerian dialect)	N	Amer Bouamar (proposed in [21])	/
D22	Kahela	Dachera	Black colour (in Algerian dialect)	N		
D23	Anonymous	Dachera	/	NS		
B1	Anonymous	Bourj El-Ghoula	/	NS	Genotype 1, proposed name: Aïn Taher *	/
B5	Anonymous	Bourj El-Ghoula	/	R		
D13	Anonymous	Dachera	/	B		
D21	Aïn Taher	Dachera	Blackberries as big as the beautiful eyes of a man (in Algerian dialect)	N		
D27	Anonymous	Dachera	/	N		
F3	Kahela seghira	El-Faidhe	Black colour and small size (in Algerian dialect)	N		
B10	Kahela	Bourj El-Ghoula	Black colour (in Algerian dialect)	N	Genotype 2, proposed name: Ineb Ganteri *	/
D5	Anonymous	Dachera	/	B		
B11	Khadhera	Bourj El-Ghoula	Green colour (in Algerian dialect)	B	Genotype 3, proposed name: Datté Ganteri *	/
D2	Datté	Dachera	Dattier (in French), or date palm fruits (in English)	N		
D8	Anonymous	Dachera	/	NS		
D9	Anonymous	Dachera	/	NS		
D19	Anonymous	Dachera	/	NS		
D26	Anonymous	Dachera	/	N		
D7	Seouikiya	Dachera	Sweet taste (in Algerian dialect)	NS	Genotype 4, proposed name: Seouikiya *	/
D25	Anonymous	Dachera	/	B	Genotype 5, proposed name: Bayedha d’El-Kantara *	/
F1	Eldjebel	El-Faidhe	The mountain	B	Genotype 6, proposed name: Ineb ElDjebel *	/

*: New identified varieties.

**Table 3:** Genetic markers (12 microsatellite loci) and parameters used to analyze their effectiveness in revealing the genetic variability of the grapevine collection. LG: linkage group; N. of obs: number of genotypes analyzed to calculate statistics; No: number of alleles; Ne: number of effective alleles, Ho: observed heterozygosity; He: expected heterozygosity; F(Null): probability of null alleles; HW: Hardy–Weinberg equilibrium; I: Shannon index; and PI: probability of identity.

Locus	LG	N. of Obs	No Alleles	Ne Alleles	Ho	He	F(Null)	HW	I	PI
VVS2	11	51	9.000	6.612	0.889	0.849	−0.047	ns	2.006	0.041
VVMD5	16	51	7.000	6.056	1.000	0.835	−0.198	ns	1.862	0.049
VVMD7	7	51	6.000	3.580	0.722	0.721	−0.002	ns	1.454	0.121
VVMD25	11	51	6.000	3.340	0.667	0.701	0.048	ns	1.385	0.140
VVMD27	5	51	5.000	3.393	0.722	0.705	−0.024	ns	1.367	0.131
VVMD28	3	51	8.000	5.023	0.944	0.801	−0.179	ns	1.816	0.064
VVMD32	4	51	7.000	3.560	0.722	0.719	−0.004	ns	1.507	0.122
VrZAG62	7	51	6.000	4.629	0.833	0.784	−0.063	ns	1.620	0.080
VrZAG79	5	51	9.000	4.378	0.722	0.772	0.064	*	1.808	0.073
VMC6E1	2	51	8.000	5.102	0.778	0.804	0.033	ns	1.769	0.067
VMC6G1	11	51	8.000	4.320	0.833	0.769	−0.084	*	1.667	0.088
VMCNG4b9	6	51	8.000	4.947	0.833	0.798	−0.044	ns	1.812	0.067
Mean			7.250	4.578	0.806	0.771			1.673	
Sum			87.000	54.941	9667	9256			20.074	
Cumulative										8.04 × 10^−14^

ns = not significant, * = *p* < 0.05.

## Data Availability

The original contributions presented in this study are included in the article/Supplementary Materials. Further inquiries can be directed to the corresponding author.

## Supplementary Materials

The following supporting information can be downloaded at: https://www.mdpi.com/article/10.3390/plants15091381/s1. Table S1: List of the geographic coordinates of the cultivar. Table S2: List of the 14 unique SSR profiles obtained at 12 SSR loci. Table S3.a: Ampelometric data of adult leaves of the novel genotypes. Tables S3.b and S3.c: Notations of quantitative and qualitative descriptors recorded for the morphological characterization of OIV.

## Abbreviations

The following abbreviations are used in this manuscript:

SSRs	Simple Sequence Repeats
*V*IVC	*Vitis* International Variety Catalogue
OIV	Organisation Internationale de la Vigne et du Vin
FAO	Food and Agriculture Organization of the United Nations
MAP	Molecular Alignment Panel
AD	Anno Domini
DNA	Deoxyribonucleic Acid
dNTP	Deoxynucleoside Triphosphate
HW	Hardy–Weinberg (Equilibrium)
PCA	Principal Component Analysis
CREA-VE	Council for Agricultural Research and Economics—Research Centre for Viticulture and Enology
PI	Probability of Identity
NJ	Neighbour-Joining
